# A homozygous *AP3D1* missense variant in patients with sensorineural hearing loss as the leading manifestation

**DOI:** 10.1007/s00439-022-02506-0

**Published:** 2022-11-29

**Authors:** Alexandra Frohne, Martin Koenighofer, Hakan Cetin, Michael Nieratschker, David T. Liu, Franco Laccone, Juergen Neesen, Stefan F. Nemec, Ursula Schwarz-Nemec, Christian Schoefer, Karen B. Avraham, Klemens Frei, Katharina Grabmeier-Pfistershammer, Bernhard Kratzer, Klaus Schmetterer, Winfried F. Pickl, Thomas Parzefall

**Affiliations:** 1 Department of Otorhinolaryngology, Head and Neck Surgery, Medical University of Vienna, Waehringer Guertel 18-20, 1090 Vienna, Austria; 2 Center of Anatomy and Cell Biology, Department for Cell and Developmental Biology, Medical University of Vienna, Vienna, Austria; 3 Department of Neurology, Medical University of Vienna, Vienna, Austria; 4 Center for Pathobiochemistry and Genetics, Institute of Medical Genetics, Medical University of Vienna, Vienna, Austria; 5 Department of Biomedical Imaging and Image-Guided Therapy, Division of Neuroradiology and Musculoskeletal Radiology, Medical University of Vienna, Vienna, Austria; 6 Department of Human Molecular Genetics and Biochemistry, Sackler Faculty of Medicine and Sagol School of Neuroscience, Tel Aviv University, Tel Aviv, Israel; 7 Center for Pathophysiology, Infectiology and Immunology, Institute of Immunology, Medical University of Vienna, Vienna, Austria; 8 Center of Translational Research, Department of Laboratory Medicine, Medical University of Vienna, Vienna, Austria

## Abstract

Loss-of-function variants in *AP3D1* have been linked to Hermansky–Pudlak syndrome (HPS) 10, a severe multisystem disorder characterized by oculocutaneous albinism, immunodeficiency, neurodevelopmental delay, hearing loss (HL), and neurological abnormalities, fatal in early childhood. Here, we report a consanguineous family who presented with presumably isolated autosomal recessive (AR) HL. Whole-exome sequencing was performed on all core family members, and selected patients were screened using array-based copy-number analysis and karyotyping. Candidate variants were validated by Sanger sequencing and assessed in silico. A homozygous, likely pathogenic p.V711I missense variant in *AP3D1* segregated with the HL. The family was characterized by thorough medical and laboratory examination. The HL was consistent across patients and accompanied by neurological manifestations in two brothers. The sole female patient was diagnosed with premature ovarian failure. Further findings, including mild neutropenia and reduced NK-cell cytotoxicity in some as well as brain alterations in all homozygous patients, were reminiscent of HPS10, though milder and lacking the characteristic albinism. Previously unrecognized, milder, isolated HL was identified in all heterozygous carriers. A protein model indicates that the variant interferes with protein–protein interactions. These results suggest that a missense variant alters inner-ear-specific functions leading to HL with mild HPS10-like symptoms of variable penetrance. Milder HL in heterozygous carriers may point towards semi-dominant inheritance of this trait. Since all previously reported HPS10 cases were pediatric, it is unknown whether the observed primary ovarian insufficiency recapitulates the subfertility in *Ap3d1*-deficient mice.

**Supplementary Information:**

The online version contains supplementary material available at 10.1007/s00439-022-02506-0.

## Introduction

Hereditary hearing loss (HL) is a heterogenous disorder that affects millions of people worldwide (Shearer et al. 1993). Genetic alterations in at least 120 genes cause isolated sensorineural HL, and more than 400 syndromes have been reported that include HL among other symptoms (Shearer et al. 1993; Van Camp 2006). Recently, a novel manifestation of Hermansky–Pudlak syndrome (HPS), referred to as subtype 10 (HPS10, MIM: 617050), has been linked to sensorineural HL by two independent reports (Ammann et al. 2016; Mohammed et al. 2019). HPS is a rare autosomal recessive disorder with an estimated worldwide prevalence of 1–2 in 1,000, 000 (Christensen et al. 2017). Ten genetically distinct subtypes (HPS1-10) have been described that are clinically heterogeneous but share oculocutaneous albinism and platelet dysfunction as the cardinal symptoms (Huizing et al. 2020). On a molecular level, the disease is caused by aberrant membrane trafficking affecting the biogenesis of secretory organelles, such as melanosomes, platelet delta granules, and other specialized, cell-type-specific compartments, depending on the disease subtype (Bowman et al. 2019). HPS2 (MIM: 608233) and HPS10 arise from genetic variation affecting the multiprotein complex adapter protein 3 (AP3) and have overlapping symptomatology. AP3 is involved in the budding and loading of vesicles and occurs in a ubiquitous and a tissue-specific, neuronal isoform (Simpson et al. 1997). Defects in the ubiquitous complex cause HPS2, which typically manifests as the characteristic oculocutaneous hypopigmentation, prolonged bleeding in many but not all patients, and recurrent infections due to immunodeficiency with neutropenia, cytotoxic T cell and NK-cell dysfunction (Dell'Angelica et al. 1999; Huizing et al. 2020). Moreover, rare instances with neurodevelopmental delay and severe courses with hemophagocytic lymphohistiocytosis and pulmonary involvement have been described (Gochuico et al. 2012; Huizing et al. 2020; Jessen et al. 2013). HPS10 is caused by variants in *AP3D1*, encoding the δ subunit of both AP3 isoforms (Kantheti et al. 1998). The resulting loss of both complexes leads to a clinical presentation that is reminiscent of HPS2 but extended by marked neurological features, such as HL, neurodevelopmental delay, and seizures (Ammann et al. 2016; Mohammed et al. 2019). The *Ap3d1*-deficient mouse model mocha mirrors multiple symptoms seen in HPS10 patients (Kantheti et al. 1998; Lane and Deol 1974; Swank et al. 1991). To date, four individuals from two families with homozygous, truncating mutations in *AP3D1* have been reported in the literature who suffered severe courses that were lethal in early childhood (Ammann et al. 2016; Mohammed et al. 2019).

Here, we report clinical and genetic findings from a consanguineous family who sought medical attention for hereditary, prelingual HL affecting six out of seven children. A comprehensive genetic analysis revealed a missense variant in *AP3D1* as the likely underlying cause. Further manifestations consistent with AP3-related disease were identified, including neutropenia, reduced NK-cell cytotoxicity, aberrant lysosomal trafficking in T cells, and neurological complaints, which varied considerably in their severity and penetrance among the affected siblings. The sole affected daughter presented with primary ovarian insufficiency, which may be part of the symptom complex but has not previously been linked to HPS10. Our results indicate that a missense variant in *AP3D1* causes human syndromic HL with HPS10-like symptoms in the absence of albinism and bleeding diathesis.

## Subjects and methods

### Patients

The family of Syrian descent was recruited at the Department for Otorhinolaryngology, Head and Neck Surgery at the Medical University of Vienna. One unaffected and six siblings affected by HL, their parents, and six related healthy controls were enrolled in the study. All core family members underwent otorhinolaryngological examination, pure-tone audiometry, and, with the exception of the sole healthy daughter, routine laboratory diagnostics. Transient-evoked (TEOAE) and distortion product otoacoustic emissions (DPOAE) testing and auditory brainstem response (ABR) audiometry were conducted bilaterally in all patients in a sound-isolated booth after they were confirmed to have a normal tympanogram. All patients further received internal and neurological examinations. Selected patients underwent German-language Goettinger speech-audiometric testing. Minors were additionally examined by a pediatric consultant. Contrast-enhanced brain magnetic resonance imaging (MRI) and high-resolution temporal bone computed tomography (CT) scans were performed in all patients affected by HL.

### Targeted genetic analysis

Chromosomal DNA was extracted from EDTA blood using a commercial kit (Invisorb, Invitek Molecular, Berlin, Germany). Whole-exome sequencing was performed for all affected family members (IV.3–5, IV.8–10), the sole healthy daughter (IV.6), the parents (III.4, III.5) and one healthy maternal aunt (III.3), and rare variants compatible with an autosomal recessive, semi-dominant, or X-linked inheritance were extracted. The data of three siblings were further screened for alternative genetic causes underlying the intellectual disabilities (IV.3 and IV.4) and primary ovarian insufficiency (IV.5), respectively, using virtual gene panels (Supplementary Tables S1 and S2). To rule out the presence of pathogenic mitochondrial variants evading variant calling due to heteroplasmy, mitochondrial genes were visually assessed as described in the Supplementary Methods. Nonsynonymous exonic variants, splice site variants, and intronic and synonymous variants with a predicted effect on splicing (assessed with SpliceAI and MaxEntScan) were considered candidates, validated and tested for segregation by PCR and Sanger sequencing (Jaganathan et al. 2019).

### Karyotyping, copy-number variation and *FMR1* trinucleotide expansion analysis

Metaphase chromosome spreads were isolated from heparin blood samples from patients IV.3 and IV.5 and evaluated by GTG banding using the karyotyping system Ikaros (MetaSystems, Altlussheim, Germany). Copy number variant (CNV) analysis was performed using a microarray (CytoSure™ Constitutional v3 + LOH). The Amplidex *FMR1* PCR kit (Asuragen, Austin, TX, USA) was used to address the possibility of a CGG trinucleotide expansion in the *FMR1* gene underlying the primary ovarian insufficiency and/or intellectual disability seen in patients IV.3 and IV.5.

### Sequence alignment, structural effect prediction, and protein model

A cross-species alignment of the peptide segment containing the *AP3D1* p.V711I variant was performed using Clustal Omega (Sievers et al. 2011). The sequences were obtained from UniProt (uniprot.org/) (UniProt. 2019). The MISSENSE3D software (missense3d.bc.ic.ac.uk) was used to predict conformational defects introduced by the p.V711I variant (based on the AP3D1 AlphaFold model AF-O14617-F1) (Ittisoponpisan et al. 2019; Jumper et al. 2021**)**. A crystal structure (PDB-ID: 4AFI) of the interaction between the VAMP7 longin domain and an AP3D1 fragment (residues 696–718) was obtained from the RCSB database (Berman et al. 2000; Kent et al. 2012), visualized and edited with PyMOL (Schrodinger, LLC. 2010. Molecular Graphics System, Version 1.8).

### Immunological workup

Peripheral blood from patients IV.3, IV.4, IV.8–10, and the parents (III.4 and III.5) was available to assess immunological function. The cytolytic activity of NK cells was tested in a standard ^51^Cr-release assay. Using flow cytometry, the T cell surface expression of the lysosomal markers CD63 and CD107a was assessed for both unstimulated peripheral blood mononuclear cells (PBMCs) and those stimulated with PMA/ionomycin for 18 h. Peripheral blood smears were examined for the presence of hypersegmented neutrophil granulocytes.

## Results

### Clinical presentation

**Fig. 1:**
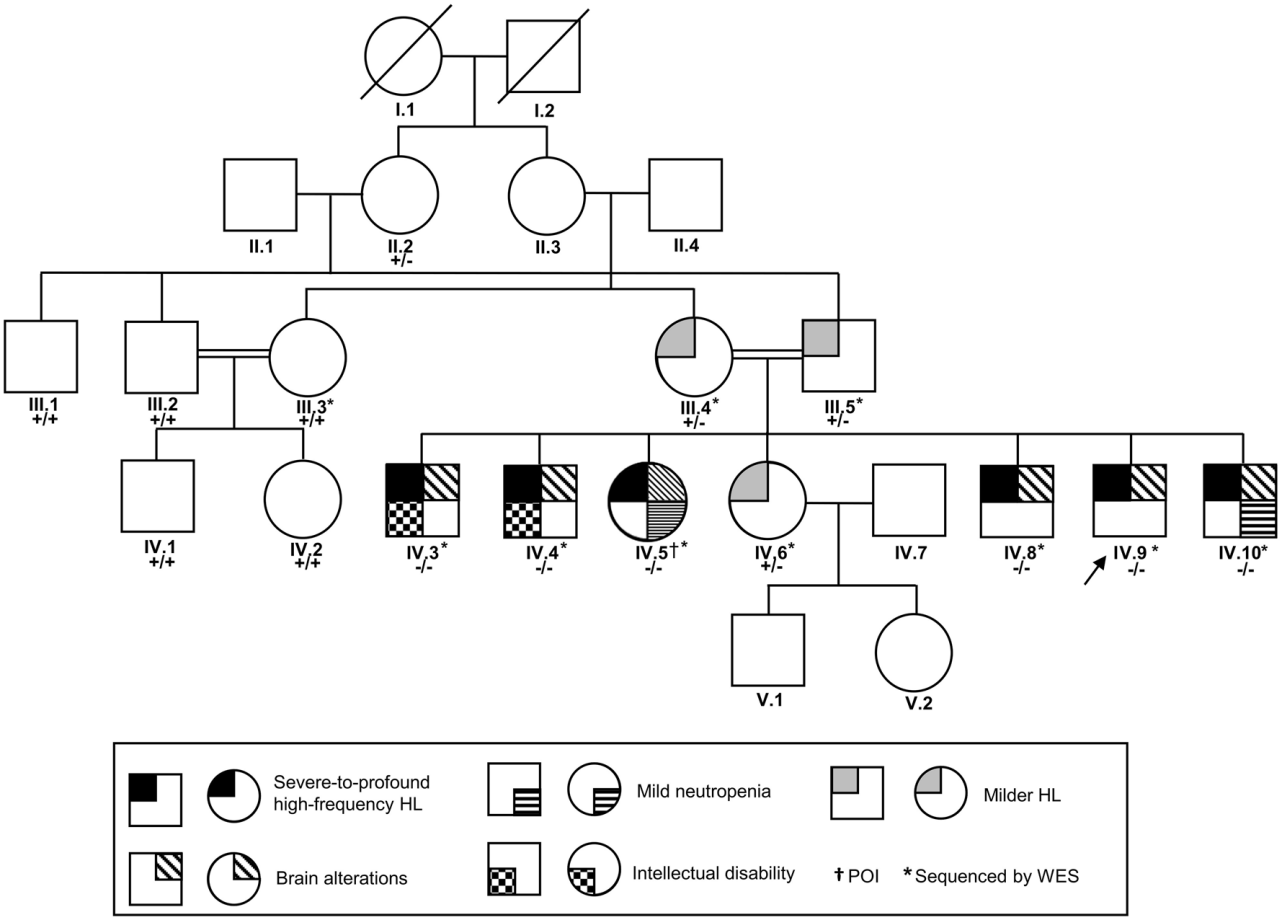
Family tree. Squares and circles represent male and female family members, respectively. The clinical phenotypes of the core family members are indicated by shades as described in the legend. Note that the neutrophil count of daughter IV.6 was not determined, and neither IV.6 nor the parents (III.3 and III.4) underwent MRI. Subjects screened by whole-exome sequencing are marked with an asterisk. The index patient is indicated by an arrow. The genotype is indicated by + (wildtype allele) and—(*AP3D1* c.2131G > A, p.V711I). *HL* Hearing loss, *POI* Premature ovarian failure, *WES* Whole-exome sequencing

**Fig. 2:**
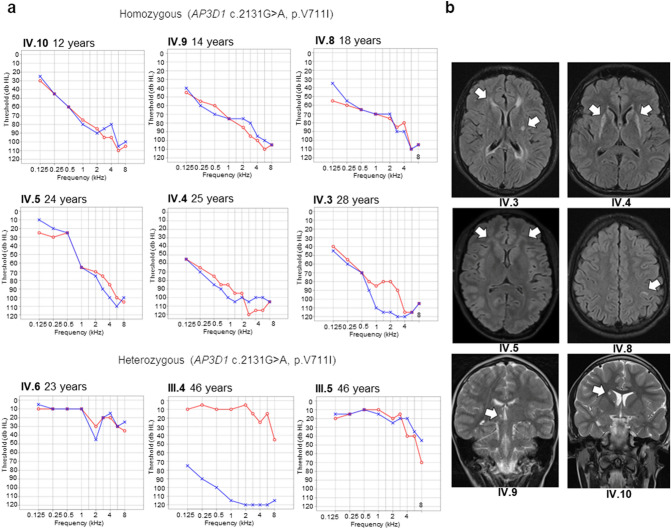
Clinical presentation. **a** Pure-tone audiograms of patients and heterozygous carriers. The unilateral HL in the mother (III.4) was acquired in childhood. Red right ear, blue left ear. **b** Cerebral signal intensity changes in six affected siblings on transverse fluid attenuated inversion recovery sequence (IV.3–IV.5, IV.8) and coronal T2-weighted (IV.9-IV.10) MR images: IV.3 (aged 27) with predominantly periventricular white matter lesions, IV.4 (aged 24) with bilateral basal ganglia lesions, IV.5 (aged 26) with subtle frontal white matter, IV.8 (aged 16) with a solitary precentral white matter lesion, IV.9 (aged 13) with a solitary thalamic lesion, and IV.10 (aged 10) with punctate basal ganglia lesions

The family under study presented with prelingual, hereditary HL affecting six out of seven siblings (Fig. 1). The audiograms of all patients showed a sloping configuration with mild-to-moderate low frequency and severe-to-profound HL in the middle and high frequencies (Fig. 2a). Aside from left unilateral severe HL of the mother, which was the consequence of an infection in childhood, the parents and daughter IV.6 had no perceived hearing difficulties. However, pure-tone audiometry revealed a decline of the high frequencies (above 7 kHz), which was milder than in the other core family members and accompanied by a mild-to-moderate threshold notch at 4–6 kHz. To address whether the HL could, at least in part, be caused by a central auditory pathology or auditory neuropathy, OAE and ABR testing were carried out. Neither TEOAEs nor DPOAEs were detectable in any of the homozygous patients tested, indicating inner ear damage. ABR responses were present at least unilaterally at supra-threshold stimulus levels in patients IV.4, IV.5, IV.8–10, and interpeak latencies were largely within the physiological range in the ears with positive ABR responses, with the exception of patient IV.10 in whom the interpeak latency between wave III to wave V was prolonged to 3.5 ms on the left side, possibly indicative of a central auditory pathway dysfunction. In patient IV.3, no clear ABR potentials could be measured up to 100 dB HL stimulus levels in both ears. An overview of the results obtained from ABR and OAE measurement is displayed in Table 1.

**Table 1:** Transient (TEOAE), distortion product otoacoustic emission (DPOAE) and auditory brainstem response (ABR) results in the study patients

Patient ID	TEOAEs		DPOAEs		ABR threshold		Interpeak latencies	
	*R*	*L*	*R*	*L*	*R*	*L*	*R*	*L*
IV.3	Refer	Refer	Refer	Refer	> 100 dB	> 100 dB	n/a	n/a
IV.4	Refer	Refer	Refer	Refer	100 dB	> 100 dB	Normal	n/a
IV.5	Refer	Refer	Refer	Refer	80 dB	80 dB	Normal	Normal
IV.8	Refer	Refer	Refer	Refer	90 dB	100 dB	Normal	Normal
IV.9	Refer	Refer	Refer	Refer	100 dB	95 dB	Normal	Normal
IV.10	Refer	Refer	Refer	Refer	> 100 dB	100 dB	n/a	III–V prolonged (3.5 ms)

*R* right ear, *L* left ear, *dB* decibel, *n/a* not applicable, *ms* milliseconds

Three of the children (IV.4, IV.6, and IV.9) had red hair and fair skin, contrasting with the black hair and dark complexion of the remaining family members, who did not co-segregate with the severe auditory phenotype. No eye-related complaints were reported in any family member apart from myopia (shortsightedness) in the father (III.5). Multiple neurological deficits were observed in the two oldest brothers (IV.3 and IV.4), including intellectual disability and ataxia. In addition, patient IV.3 presented with spasticity and mild symmetric weakness of the lower legs (Medical Research Council scale 4) associated with spastic gait. No clinical neurological abnormalities were found in any other family member, and the remaining siblings attended regular schooling. Despite the absence of neurological symptoms in the remaining siblings, MRI showed variable signal changes in the white matter, the thalamus or basal ganglia in all siblings affected by severe HL (IV.3-IV.5 and IV.8–10, Fig. 2b). CT and MRI imaging showed no abnormalities of the inner ear, temporal bone or vestibulocochlear nerve in any patient (not shown). The patient IV.3 was reported to suffer from recurrent pulmonary infections of unknown etiology (approximately twice a month). The affected daughter (IV.5) was diagnosed with primary ovarian insufficiency at the age of 25 years. All laboratory coagulation parameters, red blood count, blood chemistry, TSH, and lactate levels were normal in all patients. There were no signs of bleeding diathesis, and normal hemostasis was supported by an anamnesis with uncomplicated major surgeries in three individuals (IV.3-IV.5). The clinical manifestations in the study patients and the four previously reported HPS10 cases are summarized in Table 2.

**Table 2:** Observed manifestations in the present patients and previously reported HPS10 cases

Observed phenotype	IV.3 (m, 28 y) ^a^	IV.4 (m, 25 y) ^a^	IV.5 (f, 24 y) ^a^	IV.8 (m, 18 y) ^a^	IV.9 (m, 14 y) ^a^	IV.10 (m, 12 y) ^a^	Undef. (m, 3.5 y) ^b^	Undef. (m, 28 mo) ^c^	Undef. (f, 4 d) ^c^	Undef. (f, 6 d) ^c^
HL	+	+	+	+	+	+	+	unk	unk	unk
ID/NDD	+	+	−	−	−	−	+	+	unk	unk
Ataxia	+	+	−	−	−	−	unk	unk	unk	unk
Seizures	−	−	−	−	−	−	+	+	unk	+
Brain MRI abnormalities	+	+	+	+	+	+	+	+	unk	unk
Neutropenia	−	−	+	−	−	+	+	+	+	+
Impaired cytotoxic lymphocyte degranulation	−	+	unk	+	−	−	+	+	unk	unk
Increased CD63 and CD107a exocytosis in activated T cells	+	+	unk	+	+	+	unk	unk	unk	unk
Recurrent respiratory infections	+	−	−	−	−	−	+	+	unk	unk
Facial dysmorphism	−	−	−	−	−	−	+	+	+	+
Albinism	−	−	−	−	−	−	+	+	+	+
Flat hip acetabula	unk	unk	unk	unk	unk	unk	+	unk	unk	unk
POI	n/a	n/a	+	n/a	n/a	n/a	n/a	n/a	n/a	n/a
Remarks							Died at 3.5 y of septic pneumonia	Died at 28 mo of septic pneumonia	Died at 4 d	Died at 6 d

+ present, − not present, *f* female, *m* male, *y* years, *mo* months, *d* days, *undef.* Undefined, *HL* hearing loss, *ID* intellectual disability, *NDD* neurodevelopmental delay, *MRI* magnetic resonance imaging, *POI* primary ovarian insufficiency, *unk.* Unknown, *n/a* not applicable

^a^This study

^b^Ammann et al. (2016)

^c^Mohammed et al. (2019)

### Genetic analysis

Whole-exome analysis identified a known, biallelic NM_002386.4:c.456C > A, p.W152X nonsense variant in *MC1R* (encoding melanocortin receptor 1) as the cause of the lighter pigmentation in IV.4, IV.6, and IV.9. This variant did not segregate with the HL or the neurological phenotype.

Six rare variants segregated with the severe auditory phenotype in all persons tested by whole-exome sequencing. This included an intronic NM_000208.4:c.2683-32C > T variant in *INSR* (encoding an insulin receptor with no known role in hearing) that had no predicted effect on splicing and was, therefore, not further considered a candidate. Out of the five remaining candidates that were tested for segregation in the extended family, two variants segregated with the severe HL in an autosomal recessive (NM_016252.3:c.2448G > C, p.E816D in *BIRC6,* encoding Baculoviral IAP Repeat Containing 6) and an autosomal recessive or semi-dominant manner (NM_001261826.3:c.2131G > A, p.V711I in *AP3D1*), respectively (Table 3). No candidate genetic causes for the intellectual disability in IV.3 and IV.4 nor the primary ovarian insufficiency in IV.5 were identified in the whole-exome-based panel analyses of genes associated with the respective phenotypes (Supplementary Tables S1 and S2). Karyotyping and array-based CNV analysis showed no abnormalities, and *FMR1* trinucleotide repeats were in the normal range (≤ 45) in both tested patients (IV.3 and IV.5).

**Table 3:** Candidate variants identified in the family

Gene	Genomic coordinates (Hg19)	Variant		dbSNP	Max MAF (gnomAD 2.1.1)	Inheritance	Interpretation	CADD/REVEL
		cDNA	Protein					
*BIRC6*	g.2:32640807	c.2448G > C NM_016252.3	p.E816D NP_057336.3	rs760360568	0.00001766 (NFE)	AR	–	16/0.152
*AP3D1*	g.19:2115555	c.2131G > A NM_001261826.3	p.V711I NP_001248755.1	rs754096388	0.00003268 (SAS)	AR/SD	LP (PP1_Strong, PM2, PP4)	23/0.234
*SAFB2*	g.19:5587309	c.2807 T > C NM_014649.3	p.V936A NP_055464.1	rs565415217	0.0001911 (AFR)	*Variant not segregating*		
*LONP1*	g.19:5705913	c.1237G > A NM_004793.4	p.D413N NP_004784.2	–	–	*Variant not segregating*		
*FUT3*	g.19:5844444	c.407A > T NM_000149.4	p.N136I NP_000140.1	–	–	*Variant not segregating*		

The BIRC6 variant was not classified according to the ACMG/AMP guidelines, which are not intended for variants in genes of unknown clinical significance (Richards et al. 2015)

The variants in SAFB2, LONP1, and FUT3 were present in the homozygous state in an unaffected control (III.1)

Max MAF highest allele frequency in any of the major gnomAD populations (*SAS* South Asian, *NFE* European (Non-Finnish), *AFR* African/African-American), *AR* autosomal recessive, *SD* semi-dominant, *LP* likely pathogenic

### Sequence alignment and protein structure visualization

The p.V711I variant is located within the AP3 δ hinge domain, and the affected valine is fully conserved from human to zebrafish (Fig. 4a-c). The δ hinge engages in a protein–protein interaction with VAMP7 (vesicle-associated membrane protein 7). MISSENSE3D predicted no structural change caused by the variant. Visualization and modeling of an VAMP7 longin domain/AP3 δ construct shows that the hydrophobic AP3 δ p.711 valine side chain extends into a narrow hydrophobic groove on VAMP7, preventing an equivalent placement of the bulkier mutant isoleucine side chain (Fig. 4d) (Kent et al. 2012).

### Immunological workup

**Fig. 3:**
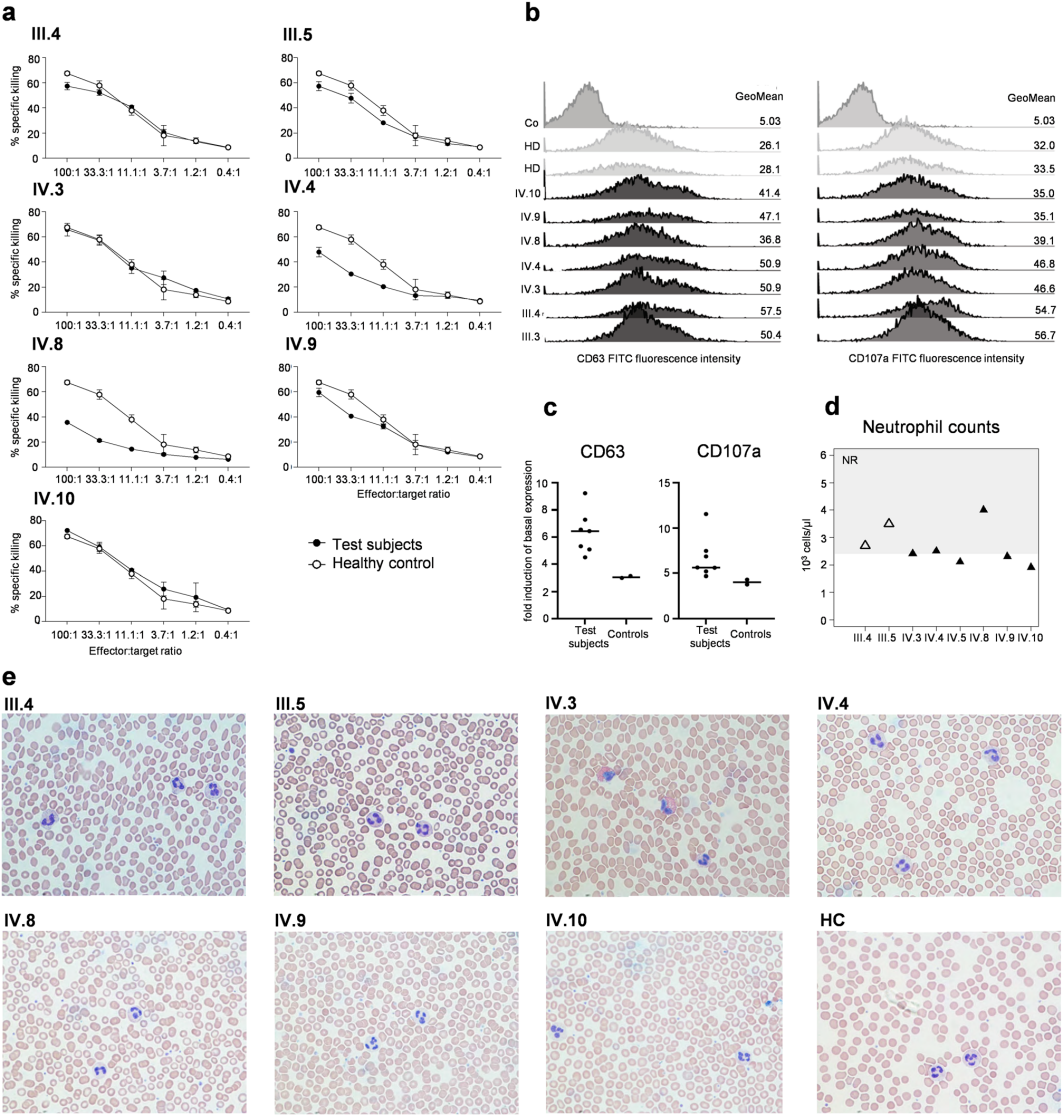
Immunological workup. **a** NK-cell assay. Constant numbers of ^51^Cr-labeled erythroleukemic K562 cells were incubated with graded numbers of patients’ peripheral blood mononuclear cells as indicated. Individual data points represent the mean of technical triplicates, whiskers show the standard deviation. Reduced cytotoxicity was evident in the patients IV.4 and IV.8. **b** Histograms showing CD63 and CD107a surface expression on T cells examined by multicolor flow cytometry. While the surface expression was comparable to the control in a resting state, levels were increased in all family members following activation. **c** CD63 (left) and CD107a (right) induction (fold-induction of basal CD63 and CD107a expression on unstimulated T cells, respectively) of all family members and two healthy controls is shown. **d** Laboratory diagnostics, neutrophil counts. Shaded and unshaded triangles indicate heterozygous and homozygous (*AP3D1* p.V711I) individuals, respectively. NR: normal range **e** Representative photographs of Wright-Giemsa-stained blood smears. Pictures show typical cell morphologies of neutrophilic granulocytes. The blood smear of patient IV.3 shows eosinophilia

NK cells of patients, their parents, and a healthy control were tested in parallel in a classical ^51^Cr-release cytotoxicity assay with K562 cells as targets. The results indicate a decrease in the activity of NK cells from patients IV.4 and IV.8, a mild, inconclusive decrease in IV.9, and normal activity in all other test persons (Fig. 3a). The observed decrease could not be explained by the variation of relative NK-cell numbers compared to the healthy control used in the assay (IV.4: 1.4-fold lower relative NK-cell counts but a fourfold decreased cytolytic activity; IV.8: 2.3-fold lower cell counts but a tenfold decreased cytolytic activity). Given that an increased CD63 surface expression has been described for cytotoxic lymphocytes from HPS2 patients (deficient in the β3A subunit of the ubiquitous AP3 complex), constitutive and phorbol-ester-induced CD3+ T cell surface expression levels of the lysosomal markers CD63 and CD107a were examined by multicolor flow cytometry, revealing normal expression in unstimulated, but increased CD63 and CD107a in activated cells from all family members compared to the healthy controls (Fig. 3b and c) (Wenham et al. 2010). Two patients (IV.5 and IV.10) had mild neutropenia that was clinically inapparent (Fig. 3d). The blood smears of all family members showed no clear signs of hypersegmentation of neutrophilic granulocytes in any family members. However, eosinophilia was immediately apparent in patient IV.3 (Fig. 3e).

## Discussion

The structure and function of intracellular compartments and specialized cell membrane domains depend on the targeted distribution of proteins and lipids. Newly synthesized components, those retrieved from the cell surface and organelles are sorted, recycled or degraded, requiring a tight and multi-layered regulation system to ensure the correct packaging into vesicles and trafficking along several routes. Biallelic variants in any of at least ten genes that encode components of this system cause HPS, a heterogeneous disorder resulting from dysfunctional organelles in variable cell types (Bowman et al. 2019; Huizing et al. 2020).

**Fig. 4:**
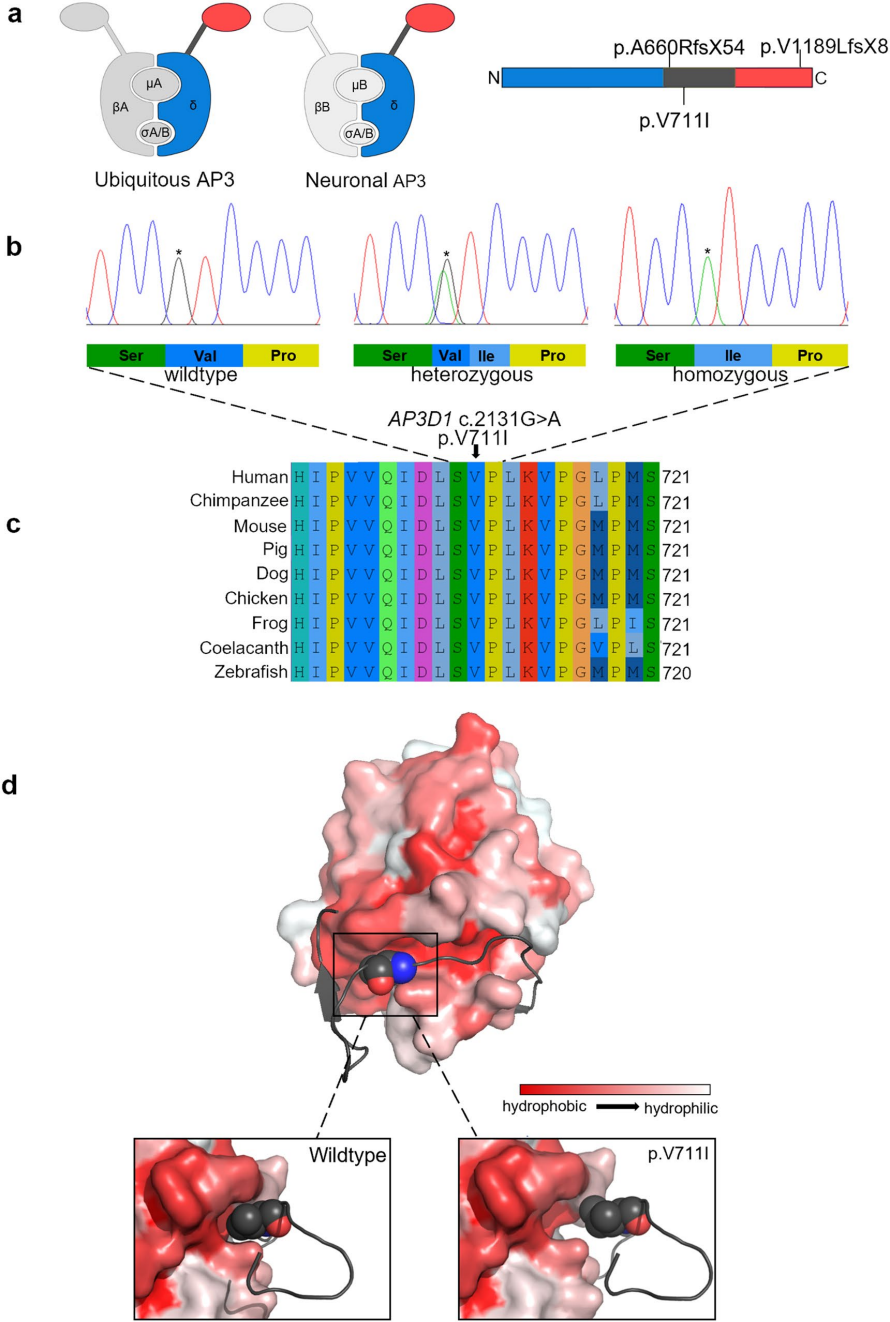
Effects of *AP3D1* genetic variation on the protein. **a** The AP3 complexes consist of two large (β and δ) and two smaller (µ and σ) units. The isoform composition of ubiquitous and neuronal AP3 are shown. The δ unit contains a ‘trunk’ (blue), ‘hinge’ (dark gray), and ‘ear’ (red) domain. The location of both reported truncating HPS10 variants and the novel p.V711I variant are indicated (Ammann et al. 2016; Mohammed et al. 2019). **b** Representative Sanger chromatograms of wildtype, heterozygous, and homozygous family members are shown, and the effect of the altered codon on the peptide sequence is indicated (**c**) The cross-species AP3 δ sequence alignment shows full conservation of p.V711 from human to zebrafish. **d** Visualization of an AP3/VAMP7 longin domain complex crystal structure (4AFI). The AP3 delta fragment containing the residues 696–719 (shown in dark gray) interacts with a hydrophobic groove of the VAMP7 longin domain (colored by degree of hydrophobicity). The hydrophobic residue of p.V711 (spheres) protrudes into the groove. Modeling of the p.V711I variant (lower right image) indicates that the bulkiness of the hydrophobic isoleucine side chain interferes with the fitting of the structures, possibly abolishing or altering the interaction. The distance between VAMP7 and AP3 was introduced for a better view on the structures and to avoid overlay

AP3 comprises four subunits designated β3, σ3, µ3 (each occurring as an A and B isoform), and δ (Simpson et al. 1997). The ubiquitously expressed AP3 complex contains the δ unit, β3A, µ3A, and either σ3 isoform, whereas the one restricted to neuronal tissues contains δ, the β3 and µ3 B-units, and σ3A or B (Fig. 4a) (Newman et al. 1995; Simpson et al. 1997). HPS2 and HPS10 are caused by genetic variants in *AP3B1*, encoding ubiquitous β3A, and *AP3D1,* encoding the δ unit of both isoforms, respectively (Huizing et al. 2020). AP3 complexes localize to the cytoplasmic surface of membranes and control the formation of vesicles destined for the late-endosomal, lysosomal or related compartments (Dell'Angelica 2009; Simpson et al. 1997). The symptomatology of AP3-related HPS reflects the set of affected organelles, including hypopigmentation caused by immature melanosomes, a bleeding tendency due to deficient platelet storage granules, immunodeficiency with neutropenia and defects of leukocyte secretory granules, and, in rare cases, pulmonary fibrosis, presumably caused by dysfunctional alveolar lamellar bodies (Bowman et al. 2019). Neuronal AP3 is further involved in synaptic vesicle trafficking (Kantheti et al. 1998; Seong et al. 2005), likely accounting for the pronounced neurological phenotype in HPS10, including neurodevelopmental delay, seizures, and HL.

In the present study, the genetic disease background of a consanguineous family was analyzed who presented with hereditary prelingual HL as the primary symptom. After the whole-exome analysis of all core family members and an unaffected aunt revealed a *AP3D1* p.V711I (2131G > A) segregating with the disease, further examinations unveiled additional neurological symptoms and immunological aberrations that were reminiscent of HPS10 but largely subclinical and showing both variable penetrance and expressivity. The oldest son suffered from bimonthly respiratory tract infections of unknown etiology. The sole affected daughter sought gynecological advice after a period of two years of an unfulfilled pregnancy wish and was diagnosed with primary ovarian insufficiency at the age of 25. Importantly, none of the family members showed signs of prolonged bleeding times nor hypopigmentation, key clinical components of previously described HPS phenotypes. Ginger hair and fair skin in some family members did not segregate with the disease and was found to be caused by a previously described *MC1R* nonsense variant (John and Ramsay 2002). Neither whole-exome screening nor testing for CNVs, large chromosomal aberrations, and fragile X syndrome yielded an alternative explanation for the neurological complaints or the primary ovarian insufficiency. The *AP3D1* missense variant was classified as likely pathogenic by the HL-specific ACMG/AMP guidelines based on the criteria PP1_Strong (segregation in at least three affected relatives), PM2 (rare in population databases) and PP4 (phenotype highly gene-specific) (Table 3) and, in view of the clinical and genetic evidence, considered the likely cause of disease (Oza et al. 2018; Richards et al. 2015).

The valine-to-isoleucine substitution is located in the hinge region of the AP3 δ unit, which connects the larger ‘trunk’ and ‘ear’ domains (Fig. 4a) and, in other adapter units with conserved domain organization, engages in protein–protein interactions (Park and Guo 2014). VAMP7 (vesicle-associated membrane protein 7), a SNARE protein mediating fusion events of late-endosomal and lysosomal membranes, has been recognized as a binding partner of the AP3 delta hinge region (Kent et al. 2012). In hippocampal neurons, VAMP7 contributes to neurotransmitter release at the terminal region (Scheuber et al. 2006), and in oligodendrocytes, it has been suggested to be involved in myelin biogenesis (Feldmann et al. 2011). The intracellular localization of VAMP7 depends on AP3 in multiple cell types (Kent et al. 2012; Scheuber et al. 2006). The AP3/VAMP7 interaction is relatively weak, probably to allow the dissociation of AP3 after vesicle formation (Kent et al. 2012). While the scores from computational prediction tools of deleteriousness point towards a possible pathogenicity of the p.V711I variant (CADD:23; REVEL: 0,234), the MISSENSE3D software predicted no structural effect on the protein. Visualization of an AP3/VAMP7 complex (Fig. 4d) demonstrates that p.V711 is located within the binding site. The hydrophobic side chain of wildtype valine is buried in a small pocket contributing to a wider hydrophobic groove on the VAMP7 longin domain, likely unable to fully contain the bulkier isoleucine side chain. Hence, the p.V711I alteration may abolish the interaction or alter its strength. Furthermore, the binding of other, yet unidentified interaction partners may be affected. Both familial cases of HPS10 reported to date were caused by truncating variants predicted to result in a complete loss of the AP3 complex (Fig. 4a), and all four patients had severe courses that, unlike the patients reported in this study, included albinism, severe immunodeficiency, microcephaly, seizures, facial dysmorphism, and were lethal in early childhood (Ammann et al. 2016; Mohammed et al. 2019). Similarly, the *Ap3d1*-deficient mocha mouse mutant, which mimics multiple aspects of human HPS10, expresses no functional AP3 (Kantheti et al. 1998; Lane and Deol 1974; Swank et al. 1991). The relatively mild phenotype seen in the family under study likely reflects the less deleterious impact of the missense variant, which appears to affect different compartments unequally. None of the patients showed signs of bleeding diathesis nor oculocutaneous hypopigmentation, even though the latter presents a highly penetrant manifestation of all HPS subtypes (Huizing et al. 2020). However, its severity fluctuates, and HPS2 and HPS10 patients typically retain varying degrees of pigmentation, indicating some redundancy of AP3 function in the biogenesis of melanosomes. Hence, a comparatively mild effect of the variant may be fully compensated by a different adapter. Alternatively, the presumed binding site containing p.V711I may be of varying relevance in different contexts. As of yet, ovarian insufficiency has been linked to neither HPS2 nor HPS10. However, the vast majority of HPS2 and all HPS10 cases described in the literature were pediatric and both male and female mice deficient in *Ap3b1* or *Ap3d1* are notoriously poor breeders (Ammann et al. 2016; Huizing et al. 2020; Lane and Deol 1974; Mohammed et al. 2019). Even though uterine hypoplasia has been observed in *Ap3b1*-deficient mice (Jing et al. 2020), the exact mechanism of reduced fertility in the *Ap3d1*-deficient mocha mice is unknown, and a possible connection with the primary ovarian insufficiency cannot be ruled out.

Strikingly, whereas the severity and/or penetrance of all other symptoms were subject to considerable fluctuations among the siblings, the auditory phenotype was fully penetrant, largely uniform, and symmetric. This may indicate a particular sensitivity of the auditory system to variation in the δ hinge region. Moreover, pure-tone audiometry revealed a similarly uniform, but milder high-frequency threshold notch between 4 and 6 kHz in all heterozygous carriers. The screening of all homozygous patients and heterozygous carriers for possible alternative causes yielded no results, indicating that the *AP3D1* variant may have a semi-dominant effect on hearing. The molecular mechanisms underlying the deterioration of *Ap3d1*-deficient cochlear tissues in mocha mice remain unknown (Lane and Deol 1974). However, a disturbed planar cell polarity due to aberrant protein trafficking has been proposed as a contributing factor (Tower-Gilchrist et al. 2019). Moreover, since an increased constitutive neurotransmitter release has been observed in hippocampal neurons of mocha mice (Scheuber et al. 2006), it is tempting to speculate that altered presynaptic dynamics may play a role in sensory hair cells where indefatigable ribbon synapses rely on steady recycling and replenishment of synaptic vesicles. A mild dysmyelination seen in mocha mice has been suggested to be driven by aberrant VAMP7 trafficking to the myelin membrane (Feldmann et al. 2011), indicating that disturbed VAMP7/AP3 binding may cause the varying patterns of brain alterations in the family. Both AP3 isoforms and VAMP7 are also expressed in cochlear hair cells (Uthaiah and Hudspeth 2010), but a role of their interaction in hearing has not been established. Although absent OAEs can be due to secondary outer hair cell demise, the OAE and ABR measurements point towards inner ear damage as the likely cause of HL in our patients. In one patient (IV.10), a prolonged wave III-V (generated from nuclei and neuronal tracts in the medulla and the pons) interpeak interval was observed, which could be indicative of a central auditory pathway dysfunction. However, this finding was restricted to one patient and must therefore be interpreted with caution. Interestingly, mice deficient in either *Ap3b1* (encoding β3A) or *Ap3b2* (encoding β3B)*,* i.e., specifically lacking ubiquitous, but not neuronal AP3, and vice versa, have largely normal hearing (informatics.jax.org, MGI submission J:205,908) (Yang et al. 2000). Other neurological symptoms such as seizures, shakiness, and hyperactivity, by contrast, are mimicked by *Ap3b2* null mice, lacking the neuronal isoform. Therefore, it appears that either AP3 isoform can compensate for the other in the cochlea of null mutants, but not in the brain. This, together with the marked impact of p.V711I on hearing, indicates that the δ unit, and the hinge region in particular, may be a key regulator in this tissue.

In conclusion, we report a family with sensorineural HL of autosomal recessive or semi-dominant inheritance, which is accompanied by a set of incompletely penetrant symptoms resembling attenuated HPS10. Further, primary ovarian insufficiency may be part of the symptom complex.

## Supplementary Information

Below is the link to the electronic supplementary material.

Supplementary file1 (XLSX 65 KB)

Supplementary file2 (DOCX 20 KB)

## Data Availability

All data relevant to the conclusions of the study are presented or referenced within the article. Any additional data that are not compromised by ethical restrictions will be made available upon request.
