# Supplementary material for: A homozygous AP3D1 missense variant in patients with sensorineural hearing loss as the leading manifestation
Source: Hum Genet. 2022 Nov 29;142(8):1077–89. doi: 10.1007/s00439-022-02506-0 (PMC10449960; doi:10.1007/s00439-022-02506-0)
Supplement: Supplementary file 2 — Supplementary file2 (DOCX 20 KB) [file 439_2022_2506_MOESM2_ESM.docx]

**Supplementary Methods

Targeted Genetic Analysis**

Chromosomal DNA was extracted from EDTA blood using a commercial kit (Invisorb, Invitek Molecular, Berlin, Germany). For the whole-exome screening of the index patient (IV.9), his parents (III.4, III.5), and unaffected sister (IV.6), libraries were prepared using the Nextera Rapid Capture Exome v1.2 kit prior to sequencing on an Illumina HiSeq 2000 device (Illumina, San Diego, CA, USA). In the remaining patients (IV.3-IV.5, IV.8, IV.10) and a healthy control (III.3), library preparation was performed with the SureSelect Human All Exon V6 kit (Agilent, Santa Clara, CA, USA), followed by sequencing using the NovaSeq 6000 system (Illumina, San Diego, CA, USA). The bioinformatic analysis in all cases included mapping with BWA-MEM, sorting with SAMtools,(Li et al. 2009) and flagging of PCR duplicates using Picard (http://broadinstitute.github.io/picard/). Variants were called with UnifiedGenotyper and HaplotypeCaller v3.8 (McKenna et al. 2010), respectively, and annotated with genetic data from NCBI RefSeq (O'Leary et al. 2016), allele frequency data from gnomAD 2.1.1 (Karczewski et al. 2020), and clinical information from ClinVar using ANNOVAR and the most recent database versions (annovar.openbioinformatics.org, accessed in February 2021) (Landrum et al. 2018). Rare (MAF (gnomAD pop_max) ≤ 0.005) variants compatible with an autosomal recessive inheritance were extracted. Moreover, to account for a milder auditory phenotype identified by pure-tone audiometry in the parents and daughter with perceived normal hearing (III.4, III.5, and IV.6), rare (≤ 0.005) variants were also analyzed under the assumption of a semi-dominant or X-linked inheritance. In the semi-dominant model, all patients with severe HL (IV.3-IV.5, IV.8-IV.10) were expected to be homozygous for a causative variant, whereas family members with milder HL were expected to be heterozygous. In the X-linked model, the male affected patients IV.3, IV.4, and IV.8-IV.10 were expected to be hemizygous, and both mildly and severely affected female family members (III.4, IV.5, and IV.6) were expected to be heterozygous for a causative variant. Nonsynonymous exonic variants, splice site variants, and intronic and synonymous variants with a predicted effect on splicing (assessed with SpliceAI and MaxEntScan) were considered candidates (Jaganathan et al. 2019). The data of three siblings were further screened for alternative genetic causes underlying the intellectual disabilities (IV.3 and IV.4) and primary ovarian insufficiency (IV.5), respectively. To this end, virtual gene panels with genes linked to the respective disease were screened for rare nonsynonymous exonic and splice site variants (Supplementary Tables S1 and S2). To rule out potential heteroplasmic mitochondrial variants, the alignments (in BAM format) of the affected family members were visualized using the Integrative Genome Viewer, and the genes MT-ND1, MT-ND2, MT-ND3, MT-ND4L, MT-ND4, MT-ND5, MT-ND6, MT-CYB, MT-CO1,MT-CO2, MT-CO3, MT-ATP6, MT-ATP8, MT-RNR2, MT-TA, MT-TR, MT-TN, MT-TD, MT-TC, MT-TE, MT-TQ, MT-TG, MT-TH, MT-TI, MT-TL1, MT-TL2, MT-TK, MT-TM, MT-TF, MT-TP, MT-TS1, MT-TS2, MT-TT, MT-TW, MT-TY, MT-TV) were visually assessed for rare (by reference to GnomAD v3) variants that occurred on more than 15% of reads.

**PCR amplification**

Candidate variants were validated and tested for segregation by PCR and Sanger sequencing. The reactions contained 50µl of 2.5mM MgCl2, 200µM of each dNTP, 100ng of DNA, 5% DMSO, and 20 pmol of each primer (designed with Primer-BLAST, ncbi.nlm.nih.gov/tools/primer-blast). The primer sequences (from 5’ to 3’) were GGAAAAGCACCAGGAGCAAC/TGTTCCTCAGTGCCCTCCTT (BIRC6, forward/reverse), TTAGGATGTTGGGGCTGCGT/TAGGGGTCGTTGGGGTCTTT (AP3D1, forward/reverse), GATTCAACAGTGCGTCTGCC/GCTGGCTGGGTTGGATGTTA (SAFB2, forward/reverse), GAGGAGTGGTTGTCCAGCAG/CACCTCACGGTCGTAGCATT (LONP1, forward/reverse), and CTCCAGGGCACTTGTATTTCAG/GATGTAGTCGGGGTGCAAGG (FUT3, forward/reverse). Amplification was performed after an initial denaturation for 5 minutes at 94°C in 35 cycles of denaturation (94°C, 30 seconds), annealing (59°C, 30 seconds), and elongation (72°C, 90 seconds), followed by a final elongation step (72°C, 10 minutes).

**CNV analysis**

Metaphase chromosome spreads were isolated from heparin blood samples from patients IV.3 and IV.5 and evaluated by GTG banding using the karyotyping system Ikaros (MetaSystems, Altlussheim, Germany). Copy number variant (CNV) analysis was performed using a microarray (CytoSure™ Constitutional v3 +LOH). The signal intensities were analyzed, background-corrected, and normalized using an InnoScan 910 microarray scanner and Mapix data acquisition and analysis software (Innopsys, Carbonne, France). The data was handled using the CytoSure™ Interpret Software (Oxford Gene Technology, Yarnton, UK). CNV calls with an absolute log-2 ratio ≥ 0.5 for deletions and log-2 ratio ≥ 0.3 for duplications extending over ≥ 10 consecutive probes were considered and annotated with data from an in-house genetic variant database (Institute of Medical Genetics, Medical University of Vienna), the Database of Genomic Variants (DGV), and dbVar (ncbi.nlm.nih.gov/dbvar) (MacDonald et al. 2014). The Amplidex *FMR1* PCR kit (Asuragen, Austin, TX, USA) was used to address the possibility of a CGG trinucleotide expansion in the *FMR1* gene underlying the primary ovarian insufficiency and/or intellectual disability seen in patients IV.3 and IV.5.

**Cross-species alignment**

The sequences were obtained from UniProt (uniprot.org/) for *Homo sapiens* (human, O14617), *Pan troglodytes* (chimpanzee, H2QEV8), *Mus musculus* (mouse, O54774), *Sus scrofa* (pig, A0A5G2R519), *Canis lupus familiaris* (dog, E2QXV5), *Gallus gallus* (chicken, A0A1D5PWB9), *Xenopus laevis* (frog, A0A1L8HWU3), *Latimeria chalumnae* (coelacanth, H3AQE4), and *Danio rerio* (zebrafish, B7ZUU8).(UniProt 2019) A crystal structure (PDB-ID: 4AFI) of the interaction between the VAMP7 longin domain and an AP3D1 fragment (residues 696-718) was obtained from the RCSB database (Berman et al. 2000; Kent et al. 2012), visualized and edited with PyMOL (Schrodinger, LLC. 2010. Molecular Graphics System, Version 1.8).

**Immunological work-up**

Peripheral blood from patients IV.3, IV.4, IV.8-IV.10, and the parents was available to assess immunological function. The cytolytic activity of NK cells was tested in a standard ^51^Cr-release assay. K562 cells (100µl of 2*10^7^/ml) were pulsed with 100 μCi of Na^51^CrO^4^ (PerkinElmer, Boston, MA) at 37°C for 1 hour. After four washes, 5x10^3^ K562 cells were added to individual wells of round bottom 96-well plates. Subsequently, titrated numbers of peripheral blood mononuclear cells (PBMCs) from test persons and an unrelated healthy control were added. Subsequently, plates were centrifuged at 100g for 5 minutes. After 5 h of incubation at 37°C, the supernatants were collected (Skarton), and the radioactivity was determined in a γ-counter (Packard). The percentage of specific release was determined as follows: [CTL-induced release (cpm) − spontaneous release (cpm)]/[maximum release (cpm) − spontaneous release (cpm)] × 100. Moreover, flow cytometric determination of CD63 and CD107a expression on T cells was performed. PBMCs were either left unstimulated or stimulated with PMA/ionomycin for 18 hours. CD63 and CD107a expression levels were detected on CD3+ T-cells by flow cytometry using CD63 FITC (H5C6, BioLegend), CD107aFITC (H4A3, BioLegend), and CD3 APC (UCHT1, BioLegend) monoclonal antibodies on a FACS Calibur flow cytometer and analyzed with the FlowJo software (both BD). Finally, peripheral blood smears stained with Wright-Giemsa solution were examined for the presence of hypersegmented neutrophil granulocytes.

**References**

O'Leary NA, Wright MW, Brister JR, Ciufo S, Haddad D, McVeigh R, Rajput B, Robbertse B, Smith-White B, Ako-Adjei D, Astashyn A, Badretdin A, Bao Y, Blinkova O, Brover V, Chetvernin V, Choi J, Cox E, Ermolaeva O, Farrell CM, Goldfarb T, Gupta T, Haft D, Hatcher E, Hlavina W, Joardar VS, Kodali VK, Li W, Maglott D, Masterson P, McGarvey KM, Murphy MR, O'Neill K, Pujar S, Rangwala SH, Rausch D, Riddick LD, Schoch C, Shkeda A, Storz SS, Sun H, Thibaud-Nissen F, Tolstoy I, Tully RE, Vatsan AR, Wallin C, Webb D, Wu W, Landrum MJ, Kimchi A, Tatusova T, DiCuccio M, Kitts P, Murphy TD, Pruitt KD. (2016) Reference sequence (RefSeq) database at NCBI: current status, taxonomic expansion, and functional annotation. Nucleic Acids Res 44: D733-45. https://doi.org/10.1093/nar/gkv1189

Karczewski KJ, Francioli LC, Tiao G, Cummings BB, Alfoldi J, Wang Q, Collins RL, Laricchia KM, Ganna A, Birnbaum DP, Gauthier LD, Brand H, Solomonson M, Watts NA, Rhodes D, Singer-Berk M, England EM, Seaby EG, Kosmicki JA, Walters RK et al. (2020) The mutational constraint spectrum quantified from variation in 141,456 humans. Nature 581: 434-443. https://doi.org/10.1038/s41586-020-2308-7

Landrum MJ, Lee JM, Benson M, Brown GR, Chao C, Chitipiralla S, Gu B, Hart J, Hoffman D, Jang W, Karapetyan K, Katz K, Liu C, Maddipatla Z, Malheiro A, McDaniel K, Ovetsky M, Riley G, Zhou G, Holmes JB et al. (2018) ClinVar: improving access to variant interpretations and supporting evidence. Nucleic Acids Res 46: D1062-D1067. https://doi.org/10.1093/nar/gkx1153

Li H, Handsaker B, Wysoker A, Fennell T, Ruan J, Homer N, Marth G, Abecasis G, Durbin R, Genome Project Data Processing S (2009) The Sequence Alignment/Map format and SAMtools. Bioinformatics 25: 2078-9. https://doi.org/10.1093/bioinformatics/btp352

McKenna A, Hanna M, Banks E, Sivachenko A, Cibulskis K, Kernytsky A, Garimella K, Altshuler D, Gabriel S, Daly M, DePristo MA (2010) The Genome Analysis Toolkit: a MapReduce framework for analyzing next-generation DNA sequencing data. Genome Res 20: 1297-303. https://doi.org/10.1101/gr.107524.110

MacDonald JR, Ziman R, Yuen RK, Feuk L, Scherer SW (2014) The Database of Genomic Variants: a curated collection of structural variation in the human genome. Nucleic Acids Res 42: D986-92. https://doi.org/10.1093/nar/gkt958

UniProt Consortium (2019) UniProt: a worldwide hub of protein knowledge. Nucleic Acids Res 47: D506-D515. https://doi.org/10.1093/nar/gky1049
